# Specific RNA Interference in *Caenorhabditis elegans* by Ingested dsRNA Expressed in *Bacillus subtilis*

**DOI:** 10.1371/journal.pone.0124508

**Published:** 2015-04-30

**Authors:** Marco Lezzerini, Koen van de Ven, Martijn Veerman, Stanley Brul, Yelena V. Budovskaya

**Affiliations:** Swammerdam Institute for Life Sciences, University of Amsterdam, Amsterdam, The Netherlands; East Carolina University, UNITED STATES

## Abstract

In nematodes, genome-wide RNAi-screening has been widely used as a rapid and efficient method to identify genes involved in the aging processes. By far the easiest way of inducing RNA interference (RNAi) in *Caenorhabditis elegans* is by feeding *Escherichia coli* that expresses specific double stranded RNA (dsRNA) to knockdown translation of targeted mRNAs. However, it has been shown that E. coli is mildly pathogenic to *C*. *elegans* and this pathogenicity might influence aging and the accuracy of the RNAi-screening during aging may as well be affected. Here, we describe a novel system that utilizes the non-pathogenic bacterium *Bacillus subtilis*, to express dsRNA and therefore eliminates the effects of bacterial pathogenicity from the genetic analysis of aging.

## Introduction

Aging is a fundamental event in all animals and is a major risk factor for most diseases. Several model organisms are currently being used to study the aging process including yeast, fruit flies, nematodes, and mice [1]. Of these, the nematode *C*. *elegans* is one of the most attractive organisms to study longevity. *C*. *elegans* normally has a relatively short lifespan of two weeks, enabling one to rapidly assess the effects of different mutations or treatments on the life expectancy. There is no somatic cell division in adult worms and a cell that is inadvertently lost is not replaced by a new cell division. Therefore, aging in *C*. *elegans* is entirely post-mitotic, reflecting the gradual loss of function in somatic cells as they grow old [2].

We know very little about how worms grow old and why they die in old age it is therefore important to understand *C*. *elegans* aging at the molecular level. The vast majority of aging research in *C*. *elegans* is based on finding and characterizing conditions or mutations that affect longevity. The RNAi technology is widely used to identify many aging related mutations on a genome wide scale. During RNAi, double-stranded RNA is introduced into *C*. *elegans* hermaphrodites leading to the rapid and sequence-specific degradation of the targeted endogenous mRNA of corresponding sequence [3]. This technique has become an extremely important tool for studying gene function *in vivo*. Initial studies with RNAi showed that injection of dsRNA in any part of the animal resulted in a robust RNAi effect in all tissues, implying that interference can cross over cellular boundaries. Later studies demonstrated that in addition to injections, RNAi can be performed by soaking worms in a solution of dsRNA, or by feeding RNAse III-deficient bacteria expressing dsRNA to *C*. *elegans* [3–5]. All three methods (injection, soaking, and feeding) can be effectively used in large-scale genome-wide studies. However, because feeding RNAi to the worms is far less labor-intensive and considerably less expensive, performing RNAi by feeding has become the preferred method for conducting genome-wide RNAi screens in aging studies. To date, scientists have identified approximately 300 *C*. *elegans* mutants that show either extended or shortened lifespan [6]. However, in any organism, including *C*. *elegans*, genetics is not the only factor influencing longevity. A great volume of recent data points towards the importance of the environment and diet in age-regulation.

Historically, *C*. *elegans* has been grown on the *E*. *coli* OP50 strain, which is a uracil auxotroph derivative of *E*. *coli* [7]. Although this strain has been widely used by *C*. *elegans* researchers since the 1970s, it remains poorly characterized. OP50 is considered to be non-pathogenic, however there is evidence of OP50 colonization and blockage of the worm intestine during old age which consequently triggers disruption of the intestinal lumen and eventually the entry of bacteria into adjacent tissues [8–10]. Therefore, we argue that *E*. *coli* might not be the best food source for *C*. *elegans*, especially in aging studies. A key question herein is: how closely does a laboratory diet of *E*. *coli* resemble the natural diet of nematodes.

In nature, *C*. *elegans* lives predominantly on decomposing plant material where it encounters a wide variety of different microbes, such as Bacilli (*B*. *subtilis* and related bacteria), Staphylococci, Streptomyces, *Micrococcus luteus*, *Comamonas* and many *Pseudomonas* species [11–13]. Interestingly, Ausubel and colleagues have found that the *C*. *elegans* lifespan is strongly affected by bacterial pathogens; worms live almost 50% longer when they are grown on a more “natural” food sources, such as *B*. *subtilis*, than when grown on *E*. *coli* [14]. However, it is not known whether this survival difference between *B*. *subtilis* grown *C*. *elegans* and *E*. *coli* fed worms is related to a difference in pathogenicity of the two types of bacteria in *C*. *elegans*. It is also unknown whether pathogenicity in general is a part of the natural aging process, as it is not known if its magnitude changes over time as the organism ages. It could be that bacteria secrete pathogenic toxins in young adults and that pathogenic damage occurring early on in life limits total lifespan or produces a mild hormesis effect that extends longevity. In this case, bacterial pathogenicity could play a role in limiting or extending lifespan, but it would not be part of the aging process *per se*. With this line of reasoning the following critical question arises: How do we distinguish the genetic mutations that extend the lifespan through up-regulation of the immune response, conferring to the organism an advantage against pathogens, from those that regulate longevity by other means unrelated to pathogenicity and the immune response?

We made a first attempt to answer this question by developing a new tool that allows us to use *B*. *subtilis* as a food source and as a shuttle organism to express dsRNA for use in RNAi experiments. Here, we demonstrate that this system works as robustly as *E*. *coli* mediated RNAi, and could be easily adapted for genome-wide applications.

## Materials and Methods

### Bacterial strains, genotypes, and growth conditions

The bacterial strain MC1061 (F^-^ *Δ(ara-leu)7697 [araD139]*_*B/r*_ *Δ(codB-lacI)3 galK16 galE15 λ* ^*-*^ *e14* ^*-*^ *mcrA0 relA1 rpsL150(strR) spoT1 mcrB1 hsdR2(r* ^*-*^ *m* ^*+*^ *))* [15] was provided by S. Brul (MBMFS, University of Amsterdam, Netherlands). The DE3 (H115) strain (*F- mcrA mcrB IN(rrnD-rrnE)1 λ- rnc14*::*Tn10)* [4] was obtained from *Caenorhabditis* Genetic Center (CGC, University of Minnesota, Minneapolis, MN, USA). The *B*. *subtilis* strain, BG322 (*ΔrncS SpR)* was obtained from D. Bechhofer (Mount Sinai School of Medicine of New York University, New York, NY, USA). This strain carries spontaneous background mutations that suppress *rncS* null lethality [16].

Plasmids were prepared in the *E*. *coli* MC1061 strain. Luria-Bertani (LB) medium was used for cultures of *B*. *subtilis* and *E*. *coli* [17]. Antibiotics were used at the following concentrations: 5 μg/ml of kanamycin for *B*. *subtilis*, and 50 μg/ml of kanamycin or 100 μg/ml of ampicillin for *E*. *coli*.

### Plasmid construction

The 1200 bp fragment containing two P*spac* promoter sequences, flanking each side of the 580 bp spacer (random DNA sequence), was synthesized *de novo* by BaseClear BV (Leiden, Netherlands) and subcloned into the pUC57 vector to generate the pYB292 vector. A BlpI-BsiWI fragment of the P*spac* driven *rodZ* gene in the pDG148-rodZ vector was replaced by the BlpI-BsiWI fragment containing the P*spac*—spacer- P*spac* to generate the pBSR feeding vector as a basis for cloning DNA fragments from genes of interest. Briefly, the 580 bp spacer can be removed by *Hind*III digestion and replaced by a gene-specific DNA fragment. The dsRNA can be produced in bacteria by transcription with RNA polymerase. pBSR also contains a *lacI* repressor to ensure suppression of dsRNA expression in the absence of iso- propyl-β-D-thiogalactopyranoside (IPTG), and provides resistance against ampicillin and kanamycin.

The GFP gene fragment was isolated after PCR amplification with genomic DNA from the SD1084 *C*. *elegans* strain, using primers 5’ GGG*AAGCTT*GATATCGGAGAAGAACTTTTCACTGGA 3’ and 5’ CCC*AAGCTT*GATATCGGTTGTCTGGTAAAAGGACAGGGCC 3’ carrying *a Hind*III restriction sites (underlined italics). The PCR product was cloned into the *Hind*III site of the pBSR vector replacing the spacer with the 750bp GFP DNA sequence to generate the pBSR-GFP vector. Plasmids were transformed into either the *E*. *coli* H115 (DE3) or the *B*. *subtilis* BG322 RNase-III-deficient strains, in which RNA polymerase can be induced by addition of IPTG to a final concentration 1 mM. The sequence of the final plasmid was verified by sequencing.

The fragments corresponding to the full-length sequence of either *daf-2* or *glp-1* genes were PCR amplified from cDNA of wild-type (*N2*/Bristol) worms, using primers: daf-2_f—GGGTTAACAAGCTTTACTGTTTGAAGACACTCTGCCA; daf-2_r—GGGTTAACAAGCTTAAACTGTGCTACACGAAAACGAT; glp-1_f—GGGTTAACAAGCTTATTGGACCGGAATGGTATGA; glp-1_r—GGGTTAACAAGCTTTGGGAGGACAAGAAACATCC. Both fragments were subsequently cloned into pBSR vector as described above. The 1600bp corresponding to unc-62 gene fragment was subcloned as EcoRV-EcoRV fragment obtained from L4440-unc-62 plasmid (Ahringer’s RNAi library) [4]. All constructs were verified by sequencing.

### Preparation and use of chemically competent cells

Efficient chemically competent H115 (DE3) and MC1061 *E*. *coli* bacteria were prepared and used as described in [18].

Chemically competent BG322 *B*. *subtilis* bacteria were prepared as follows: one colony from LB plates was incubated overnight at 37°C, 250 rpm. The coming day, 1 ml culture was added to 10 ml GCHE medium and incubated for 3 hours at 37°C, 250 rpm. Next, 11 ml of pre-warmed GE medium were added and continued the incubation for other 2 hours. Competent cells so prepared were then immediately used or stored at −80°C for later use.

### Transformation Procedure

100**μ**l of freshly made (or thawed in ice) competent cells were mixed with up to 10**μ**l (0.7–1.5**μ**g) of plasmid. Cells were incubated for 30 minutes at 37°C, 250 rpm. 300 μl of pre-warmed LB medium were added and kept the incubation at 37°C, 250 rpm for other 45 minutes. The cells were then plated on LB plates containing 5μg/μl kanamycin and put at 37°C overnight.

### *C*. *elegans* strains and growth conditions

*C*. *elegans* strains were maintained and handled as described previously [7]. The strains used in this study are: N2 (wild type *C*. *elegans* strain from Bristol); SD1084 (*gaIs148* [*ges-1p*::FLAG::*pab-1* + *sur-5*::GFP]); CF1553 (muIs84 [(pAD76) *sod-3p*::GFP + *rol-6*]) and TJ356 (zIs356 [*daf-16p*::*daf-16a/b*::GFP + *rol-6*])

### Analysis of Lifespan in *C*. *elegans*

Lifespan analyses were conducted at 15°C or 20°C as previously described [19]. Briefly, worms were synchronized by hypochlorite treatment. At least 70 N2 worms were grown on *E*. *coli* OP50 from L1 until the L4 stage and then transferred, as one-day-old young adults, onto FUdR-NGM plates freshly seeded with different bacterial strains. The population was checked for dead worms approximately every other day during the adulthood and p-values were calculated using the log-rank (Mantel-Cox) method [20] in Prism 6; GraphPad software.

### RNA-interference (RNAi) experiments

DE3 or BG322 bacteria transformed with RNAi vectors expressing dsRNA of the genes of interest were grown at 37°C in LB with either 100 μg/ml ampicillin for *E*. *coli*, or 5 μg/ml kanamycin for *B*. *subtilis*, then seeded onto NGM-ampicillin/kanamycin plates supplemented with 2 mM IPTG. One-day-old young adult worms were added to the plates and GFP fluorescence was measured 24 or 48 hours later.

### RNA extraction and qRT-PCR Analysis

The animals were synchronized by hypochlorite treatment. The animals were exposed to RNAi treatment (control/*gfp* RNAi; *E*. *coli*/*B*. *subtilis*) for 2 days at 20°C until L4/young adult stage. Worms were harvested and total RNA was extracted using Trizol and Direct-zol RNA MiniPrep Kit (Zymo Research, Cat #R2052) according to the manufacturer’s instructions. 80 ng of total RNA per reaction were used for quantitative real-time PCR (qRT-PCR) analysis, performed in triplicate with Power SYBR Green RNA-to-Ct 1-Step Kit (Applied Biosystems, Foster City, CA, USA, Part #4389986), according to the manufacturer’s instructions. Vector pD4H1 containing mCherry sequence was used to build a standard curve for absolute quantification. The relative fold change of *daf-2* and *glp-1* mRNA expression was normalized to *tba-1* level of expression. The primers used:

mCherry-Fw AGGGTTTTAAGTGGGAACGC

mCherry-Rev GCATAACAGGTCCATCCGAG

GFP Fw GGAGAAGAACTTTTCACTGGA

GFP Rev CCGAACTGTTTAAACTTACGT

*daf-2* Fw CTGGTCAGAGAATGGTCAACTG

*daf-2* Rev CACGTAGATGCGGAAAAGTG

*glp-1* Fw GGCTATGGAGGTCCTGACTG

*glp-1* Rev TTCTGCGCATTCATTTTGAG

*unc-62* FW GGAGTCTATTTATGCCCATCC A

*unc-62* Rev CAAATTCGTTGAGATCATCTTTG

*tba-1* Fw TCAACACTGCCATCGCCGCC

*tba-1* Rev TCCAAGCGAGACCAGGCTTCAG

### RNAi-induced phenotypes assays

*unc-62 RNAi* [21]: L4 animals fed with control or *unc-62*(RNAi) were allowed to lay eggs for 24h at 20°C. Next day parents were removed and the plates left for another day at 20°C. Embryonic lethality was scored by counting unhatched embryos. To check larval lethality, surviving larvae were followed until they died (larval lethal phenotype), or became fertile adults (no effect).

*daf-2 RNAi* [22]: fertile animals were allowed to lay eggs at 20°C and then the plates were shifted at 27°C. The dauer phenotype was scored 48h later, by treating the animals with a 1% SDS solution for 15–20 minutes and counting the survived animals as dauer larvae.

*glp-1 RNAi* [23]:L4 animals were allowed to lay eggs for 24h at 20°C and number of progeny at day one was counted.

### Imaging

Images of 20–25 live animals anesthetized with 1 mM levamisole, were captured using the Zeiss Axiovert 40CFL microscope equipped with an Axiovert digital camera, and analyzed using ImageJ software. Representative images were assembled using Adobe Illustrator.

## Results and Discussion

### *Bacillus* *subtilis* strain selection and construction of the RNAi vector

Performing RNAi by feeding requires cloning a DNA fragment corresponding to the gene of interest into a vector for dsRNA expression under an inducible promoter. This vector could then be transformed into an RNase III-deficient strain of *B*. *subtilis* where expression of dsRNA can be induced. It has been shown that RNase III deficiency significantly improves the efficiency of RNAi by feeding, likely because the dsRNA fragments produced are more stable in the bacteria [24]. We started by putting together these two important components for RNAi in *B*. *subtilis*.

**Fig 1:**
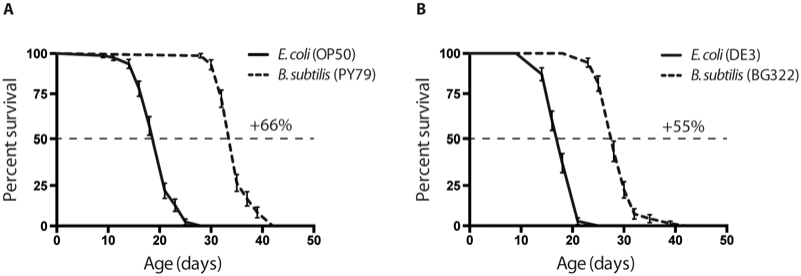
Effects of various *E*. *coli* and *B*. *subtilis* strains on longevity. (A) Worms fed on wild type *B*. *subtilis* strain (PY79) live 65% longer compared to wild type, standard laboratory food *E*. *coli* strain (OP50). (B) Worms fed on RNase III-null *B*. *subtilis* (BG322) strain live 55% longer compared to DE3, *E*. *coli* strain used for expressing stable dsRNA for RNA interference. Median survival on: OP50 = 21 days, PY79 = 35 days DE3 = 18 days, BG322 = 28 days. Age refers to days of adulthood. Three biological replicates were observed for each experiment (n = 70–80 worms per experiment); error bars indicate Standard Error. In both graphs p < 0.0001.

The first step in developing a *B*. *subtilis* strain that is useable for RNAi was to ensure that a vector and eventual dsRNA could be expressed without perturbing host strain viability. *B*. *subtilis* contains the *rncS* gene, which encodes the sole endoribonuclease that specifically cleaves dsRNA. This is an essential gene and Δ*rncS* strains are not non-viable. Fortunately, a few years ago, the D. Bechhofer’s group was able to isolate a rare Δ*rncS* strain (BG322) that carries spontaneous background mutations which suppress Δ*rncS* lethality but not the RNase III processing defects of rRNAs or prescRNAs [16]. We wanted to check whether this spontaneous mutation (or the lack of the RNase III gene) would introduce a potential toxic effect that could affect the worm’s lifespan. In this case worms grown on the BG322 strain should live as long as, or at least similarly to, the ones grown on wild type PY79 *B*. *subtilis* bacteria previously used in longevity studies [25]. We performed a lifespan analysis of the wild type *C*. *elegans* strain, N2, on the traditional *E*. *coli* strains, OP50 and the strain for RNAi analysis (H115 (DE3)), and on the *B*. *subtilis* strains, wild type (PY79) and BG322. Our results show that worms grown on *B*. *subtilis* live, on average, 50% longer than on *E*. *coli* (Fig 1). Both the worms fed on the *E*. *coli* and *B*. *subtilis* RNAi strains live slightly shorter than their wild type counter parts, suggesting that the mutation in the RNase III gene might affect bacterial physiology and, as a consequence, *C*. *elegans* lifespan. However, this effect is very small compared to the effect that both *B*. *subtilis* strains have on overall longevity. In addition we perform a wide variety of food switching experiments, and found that as soon as *C*. *elegans* grow on non-pathogenic bacteria in adulthood (post-development), they would live longer (S1 Fig and S1 Table). Worms grown on *E*. *coli* from *L1* to adulthood before being transferred to *B*. *subtilis* live as long as worms that lived on *B*. *subtilis* from *L1* larvae stage. It implies that developing worms either are not affected by pathogenicity of *E*. *coli*, or manage to efficiently fight the infection.

**Fig 2:**
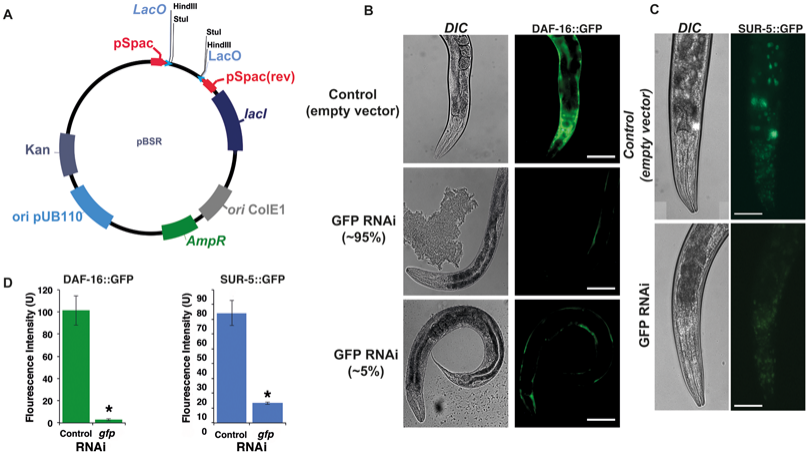
Genetic interference following ingestion of anti-GFP dsRNA-expressing *B*. *subtilis* by *C*. *elegans*. (A) Physical map of the pBSR vector. The DNA sequence corresponding to dsRNA of interest was cloned between flanking copies of the P*spac* promoter to replace the spacer. *B*. *subtilis* strain BG322 was used as a host. GFP-expressing *C*. *elegans* strains TJ356 (B) and SD1084 (C) were fed on BG322 strains transformed with original pBSR vector and on bacteria expressing dsRNA corresponding to the *gfp* coding region. Under these conditions, 95% of the animals showed dramatic decrease in GFP expression after 24 hours of feeding. DAF-16::GFP and *SUR-5*::GFP expression is significantly decreased in the GFP (RNAi) treated animals. RNAi was induced starting at L4 larvae stage by feeding worms *B*. *subtilis* bacteria expressing dsRNA against GFP. GFP expression was measured at day 2 of adulthood. The y-axis denotes GFP expression (arbitrary units). Average expression and Standard Error from 20 animals are shown. *-p-value < 0.001 (t-test p-values). Scale bar = 100 μm.

Second, we designed a vector, pBSR (*B* *acillus* *s* *ubtilis* RNAi), for bidirectional transcription of the desired dsRNAs. This vector has the following properties: (i) It carries two origins of replication and two selection markers: the ColE1 replication sequence and a β-lactamase gene (AmpR) for amplification and selection in *E*. *coli*; the pUB110 origin and kanamycin resistance gene, for amplification and selection in *B*. *subtilis* [26]. (ii) The vector has two bidirectional isopropyl-β-D-thiogalactopyranoside (IPTG)-inducible P*spac* promoters. The P*spac* promoter is a hybrid of the phage SPO1 promoter and one of the three lac operators, designated “O1” [27,28]. Separating these two promoters there is a 500 bp spacer, which is flanked by a *Hind*III and a *Sal*I restriction sites to facilitate the cloning steps. (iii) The vector carries the *lac*I gene, encoding the *E*. *coli Lac* repressor under control of the penicillinase promoter (P*pen*) of *Bacillus licheniformis* that prompts *lacI* to be constitutively expressed *in B*. *subtilis* [27]. The P*spac* promoter is repressed by *lacI* and consequently can only be induced when IPTG is present [27] (Fig 2A).

### *B*. *subtilis*-induced RNAi is effective in inhibiting a GFP transgene

Next, we evaluated the effectiveness of dsRNA expressed from the pBSR vector to interfere with green fluorescent protein (GFP) transgene expression. For these experiments, we used two GFP-expressing strains: the SD1084, expressing the nuclear localized SUR-5::GFP translational fusion, and the TJ356 strain, expressing the predominantly cytoplasmic DAF-16::GFP translational fusion. The *sur-5* gene encodes a protein with a high similarity to the *Homo sapiens* Acetoacetyl-coenzyme A synthetase, and it is broadly expressed in *C*. *elegans* [29]. *daf-16* encodes the sole *C*. *elegans* forkhead box O (FOXO) homologue that acts in the insulin/IGF-1-mediated signaling (IIS) pathway which regulates development, dauer formation, longevity, fat metabolism, stress response, and innate immunity [30]. When these animals expressing the GFP transgene were fed *B*. *subtilis* bacteria expressing dsRNA corresponding to the GFP reporters [3,31], a decrease in GFP fluorescence was observed in about 90% of the worms in the population (Fig 2B–2D, and S2A Fig). GFP expression levels were reduced to 3% of the original expression level in the DAF-16::GFP worms, and were lowered almost 6 times in SUR-5::GFP animals. As on *E*. *coli*, *B*. *subtilis* expressing dsRNA was not able to affect neuronal GFP expression (Fig 2). These results suggest that dsRNA against GFP is produced by *B*. *subtilis* and is capable of an effective gene-specific knock-down of GFP expression in *C*. *elegans* upon ingestion.

### Effect of *B*. *subtilis* induced RNAi against known regulators of longevity on DAF-16 nuclear localization

To further validate the effectiveness of *B*. *subtilis* induced RNAi, we created plasmids designed to produce dsRNAs corresponding to three endogenous *C*. *elegans* genes that were found to be involved in the modulation of longevity.

Our first choice was the *daf-2* gene, which encodes the insulin/IGF-1 signaling (IIS) receptor ortholog in *C*. *elegans*. Mutant worms with a defective *daf-2* function have been shown to live twice as long when compared to wild type animals [19] and this phenotype largely depends on the function of the *daf-16*/FOXO transcription factor: loss-of-function mutations in *daf-16* suppress the longevity phenotype of *daf-2* mutants, showing that *daf-2* acts upstream of the *daf-16* FOXO transcription factor repressing its activity [32].

The second gene chosen was *glp-1* which encodes a Notch family receptor, essential for germ line development and longevity [33]. RNAi inactivation of the *glp-1* gene leads to a significant lifespan extension, and also this effect is dependent on the *daf-16*/FOXO transcription factor [34].

Finally, we chose the *unc-62* gene. *unc-62* is an important developmental regulator, and an ortholog of the Drosophila Homothorax gene. *unc-62* directly binds to the promoter of many age-regulated genes and in this way modulates lifespan [35]. Although *unc-62* is expressed in diverse tissues, its functions in the intestine play a particularly important role in modulating lifespan, as an intestine-specific knockdown of *unc-62* by RNAi increases lifespan by 30% when compared to wild type worms. Even in this case, it has been shown that *unc-62* (RNAi) leads to an activation of *daf-16*, which is required for the extended lifespan in the treated worms [35].

Although all of these three genes play a different role in the worm physiology, and are expressed in different tissues, mutation analysis of each of these showed a significant lifespan extension when worms are grown on *E*. *coli*; and the long life of these mutants depends on the activity of *daf-16*/FOXO transcription factor. Therefore, we set out to test whether dsRNAs corresponding to each of these genes expressed in *B*. *subtilis* can influence *daf-16* and hence *C*. *elegans* lifespan.

Fragments corresponding to *daf-2*, *glp-1*, or *unc-62* were cloned into the pBSR vector (see Materials and Methods). Clones that tested positive by PCR analysis and restriction digestion were transformed into the *B*. *subtilis* BG322 strain for further analysis. The efficiency of RNAi-mediated down-regulation of these genes was tested by qRT-PCR and by scoring previously described phenotypes. First, the *daf-2* (RNAi) and *glp-1* (RNAi) treatments in wild type worms lead to approximately 20 to 40% decrease in the corresponding mRNA levels (S2B Fig). The *daf-2* (RNAi) and *glp-1* (RNAi) treatments in RNAi-sensitive (NL2099) worms lead to approximately 40 to 50% decrease in the corresponding mRNA levels (data not shown). Unfortunately we were unable to detect a significant reduction in *unc-62* level of expression. Second, we were able to score several known phenotypic effects on the treated animals (S2C Fig)[21–23]. For example, similar to *E*. *coli* treatments, *daf-2* (RNAi) treatment using *B*. *subtilis* in combination with increased temperature (27^°^C) forces wild-type worms to enter into the dauer stage and the *glp-1* (RNAi) treatment caused mild sterility, whereas the *unc-62* (RNAi) treatment led to embryonic and larval lethal phenotypes. Furthermore, these effects proved to be more prominent when the assays were carried out using the NL2099 strain.

**Fig 3:**
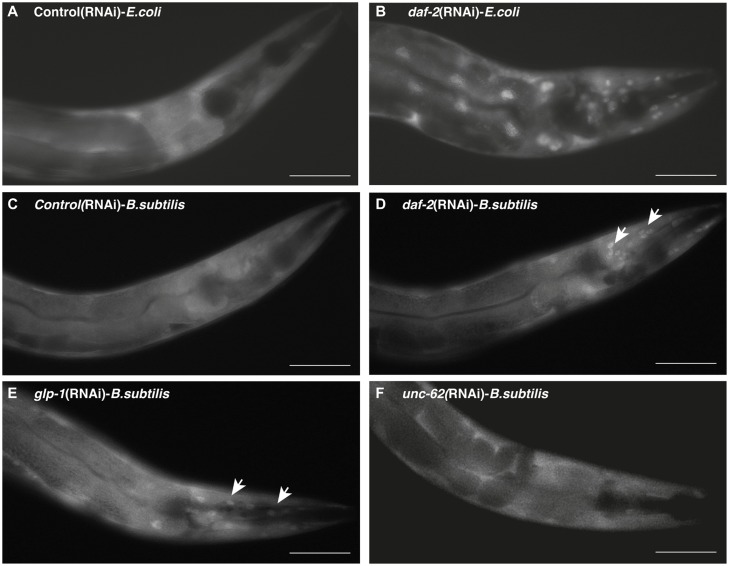
DAF-16/FOXO activated in *daf-2* (RNAi) and *glp-1*(RNAi) mutants when grown on *E*. *coli* or *B*. *subtilis*. Green fluorescent protein images of adult TJ356 (Is*daf-16*::GFP) transgenic animals shown at 40X. (A) Images of an animal raised on *E*. *coli* expressing empty vector for RNA interference. (B) Images of an animal grown on *E*. *coli* and exposed to *E*. *coli* expressing *daf-2* dsRNA for 48 hours post development. Note strong nuclear localization in most tissues, including the intestine. (C) Image of an animal grown on *B*. *subtilis* expressing the empty vector for RNA interference. Images of animals fed *B*. *subtilis* expressing either (D) *daf-2* dsRNA, (E) *glp-1* dsRNA, or (F) *unc-62* dsRNA for 48 hours post development. Note weak activation of DAF-16/FOXO in the head area upon treatment with daf-2 and glp-1 dsRNA. Scale bar = 100μm

To test the effectiveness of dsRNA against *daf-2*, *glp-1*, and *unc-62* in regulation of *daf-16* localization, we used the reporter strain TJ356, a DAF-16::GFP translational fusion strain. When the IIS pathway is active, DAF-16 is inactive and sequestered in the cytoplasm. However, upon inactivation of insulin signaling, DAF-16 becomes active and translocates to the nucleus. Multiple publications have shown that when worms are fed on *E*. *coli*, a down-regulation of *daf-2* or *glp-1* leads to DAF-16/FOXO nuclear translocation in various tissues, whereas *unc-62* (RNAi) treatment didn’t lead to any nuclear localization of DAF-16/FOXO (Fig 3A and 3B and [34–36]). Interestingly, 90% of worms grown on the *daf-2* (RNAi) *B*. *subtilis* or 83% of worms grown on the *glp-1* (RNAi) *B*. *subtilis* exhibited DAF-16 nuclear localization, but mainly in cells located in the head region (Fig 3C–3E, S2D Fig). In contrast, 95.5% of worms grown on the *daf-2* (RNAi) *E*. *coli* exhibit strong nuclear localization in the head and intestinal cells. When the worms were grown on *B*. *subtilis unc-62* (RNAi), we could not see any DAF-16 nuclear localization, neither in the head, nor in the intestine region (Fig 3F, S2D Fig). One possible explanation for this difference can be that the dsRNA is not optimally ingested by *C*. *elegans* grown on *B*. *subtilis* dsRNA expressing cells because the worm cannot efficiently grind and digest the *B*. *subtilis* vegetative cells due to the thick cell wall. Alternatively, the *B*. *subtilis* strain we used might form spores in our experimental conditions, preventing the worms from digesting these [37].

The first possibility, that *C*. *elegans* cannot digest *B*. *subtilis* vegetative cells, can be discarded because RNAi against GFP works very well in most *C*. *elegans* cells when *B*. *subtilis* is used as host strain. We indeed observed an almost complete knock-down of both the cytoplasmic and the nuclear expressed GFP targets. Moreover, no intact bacterial cells were observed in the worms’ intestine when they were fed *E*. *coli* or *B*. *subtilis* vegetative cells, suggesting the complete destruction of these bacteria by the worm’s grinder (data not shown; previously shown in [37,38]). Next, we tested the *B*. *subtilis* strain BG322 ability to sporulate on NGM plates, but no spores could be found. In addition to circumvent this possible problem, we transferred the worms every 2–3 days onto fresh dsRNA expressing BG322 strains. However, the questions still remains, why *daf-2*(RNAi) or *glp-1*(RNAi) in *B*. *subtilis* strain is less effective to activate DAF-16 nuclear translocation, then the same clone expressed in *E*. *coli*? Based on our observations, we can suggest, that *B*. *subtilis* induced RNAi causes milder effect on down-regulation of target mRNA levels than *E*. *coli* induced RNAi treatment. Another possibility is that *B*. *subtilis* is less pathogenic than *E*. *coli*. Therefore, the knock-down of either *daf-2* or *glp-1* gene is not sufficient to drive strong nuclear localization and activation of DAF-16/FOXO. More detailed studies are needed to validate or refute these alternatives.

### DAF-16 activity in worms exposed to *B*. *subtilis* induced RNAi against longevity regulators

We next analyzed the activity of DAF-16 by measuring the expression of *sod-3* gene, a well-known DAF-16 direct target, which encodes one of the *C*. *elegans* manganese-superoxide dismutases involved in oxidative stress response and longevity [36,39,40]. To this purpose, we used the CF1553 strain expressing a *sod-3*::GFP transcriptional fusion. We chose this gene because our studies as well as recent publications have demonstrated that its expression highly correlates with *C*. *elegans* lifespan and can be used as a predictor of remaining life in the worm [25,35,41].

**Fig 4:**
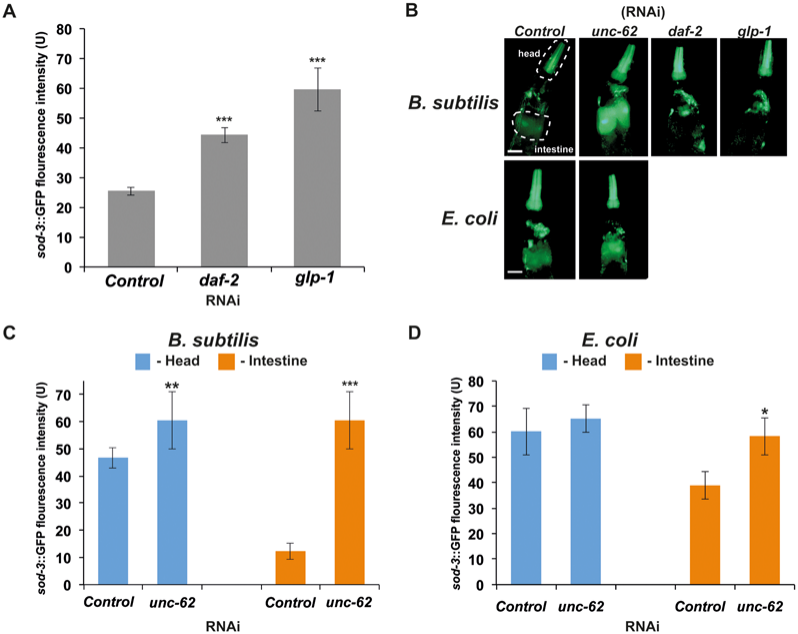
Consequences of *daf-2* (RNAi), *glp-1* (RNAi), and *unc-62* (RNAi) on *sod-3* expression. (A) *sod-3*::GFP expression is increased in *daf-2* (RNAi) and *glp-1* (RNAi) treated animals. RNAi was induced at L4 larval stage by feeding worms *B*. *subtilis* expressing dsRNA. *sod-3*::GFP expression was measured at day 2 of adulthood. The y-axis denotes GFP expression (arbitrary units). Average expression and Standard Error from 20 animals are shown. (B) Representative pictures of expression of *sod-3*::GFP at day 2 of adulthood in *unc-62* (RNAi), *daf-2* (RNAi) and *glp-1* (RNAi) treated animals. The dashed lines indicate areas where fluorescence intensity was quantified to measure GFP intensity in the head or intestine (dashed lines in representative pictures (B) indicate the areas used in quantifications) upon RNAi treatment. *sod-3*::GFP expression is increased in the intestine of *unc-62* (RNAi) treated animals. RNAi was induced starting at L4 larvae stage by feeding worms (C) *B*. *subtilis* or "(D) *E*. *coli* bacteria expressing dsRNA. *-p-value < 0.05, **- p-value >< 0.01, and **-p-value ><0.001 (t-test p-values). Scale bar = 50μm.

When *C*. *elegans* grows on either *B*. *subtilis* or *E*. *coli* as a food source, *sod-3* expression decreases with age (S3 Fig). When young adults expressing the *sod-3*::GFP transgene were fed *B*. *subtilis* bacteria expressing dsRNA corresponding to either *daf-2* or *glp-1* genes, an increase in GFP fluorescence was observed mainly in the head region (pharynx, head hypodermis, and neurons) (Fig 4A and 4B). These results are consistent with the DAF-16::GFP nuclear localization observed in the same region of the worm (Fig 3D and 3E).

On *B*. *subtilis unc-62* (RNAi) however, the expression of *sod-3* significantly increased in the head and in the intestine region of the worm (Fig 4C). These expression levels remained high throughout the *C*. *elegans* life (S3B Fig), whereas on *E*. *coli unc-62* (RNAi), the worms showed an increase in *sod-3*::GFP expression mainly in the intestine (Fig 4D). This is a very puzzling phenotype since we were unable to see any nuclear localization of the DAF-16/FOXO in *B*. *subtilis unc-62* (RNAi) (Fig 3F). These data suggest that this increased expression of *sod-3* may not entirely be dependent on DAF-16 activity. There are two other alternative explanations for this observation. First, expression of the *unc-62* in worms grown on *B*. *subtilis* is much lower then in worms grown on *E*. *coli*. In this case using the *B*. *subtilis* as a food source gives as the same benefits as *unc-62* down-regulation. The second, *unc-62* is expressed at regular level, but because *B*. *subtilis* is non-pathogenic, and does not cause any damage to intestine (see next section) down regulation of this gene does not provide additional benefit on worm life span. The further analysis of *unc-62* expression behavior on different food sources is beyond the scope of this paper. More work is needed to investigate this observation further.

### Worms grown on *B*. *subtilis* accumulate less lipofuscin

Lipofuscin is a fluorescent aggregate of lipids and damaged proteins which accumulates with age and it is often used as a biomarker for aging [42–45]. It was previously demonstrated that accumulation of lipofuscin is accelerated, as the worms grow older [9]. Interestingly, in long-lived mutants lipofuscin accumulates at low rates and has been associated more with physiological rather than chronological life span [46]. Here, we would like to report a quite intriguing phenotype of lipofuscin accumulation when worms were grown on *unc-62* (RNAi) expressed in either *E*. *coli* or *B*. *subtilis*.

First, we observed very little lipofuscin accumulation in the gut of the worms grown on *B*. *subtilis* compared to worms grown on *E*. *coli* (S4 Fig). This result corroborates the previous observation that *B*. *subtilis* could be a healthier diet for *C*. *elegans* grown under laboratory conditions. As the intestine is the primary site for the response to a pathogenic infection [8], one possible explanation for this effect could be that *B*. *subtilis* is less pathogenic in this organ and therefore causes less damage to proteins and lipids residing in the worm’s intestine.

Second, we observed a significant reduction in intestinally accumulated lipofuscin in *unc-62* (RNAi) mutant worms grown on *E*. *coli* (S4 Fig). It has been previously shown, that UNC-62 regulates many intestine specific age-regulated genes, including all six vitellogenin encoding genes [35] that are prone to damage and aggregation with age. In long-lived *unc-62* (RNAi) mutants grown on *E*. *coli*, expression of vitellogenin is reduced, and this might contribute to low overall gut/lipofuscin auto-fluorescence and longer life. On *B*. *subtilis* however, we observed quite the opposite effects of the *unc-62* (RNAi) knockdown on the lipofuscin accumulation in the gut of the worms compare to empty vector control bacteria. These mutant worms show almost seven fold increase in accumulation of gut autofluorescence (S4 Fig). These results show that *unc-62* is expressed in worms grown on *B*. *subtilis*, and its inactivation leads to increase in accumulations of autofluourscent proteins in the gut. Several interesting questions were raised by these observations. Is the lipofuscin composition different when worms are using *B*. *subtilis* as a food source? What kind of gene expression and metabolic changes accompany *B*. *subtilis* promoted health span extension? More experiments are needed to address these issues. It is entirely possible that we can use *B*. *subtilis* and *C*. *elegans* to identify a novel mechanism of longevity in worms by examining the beneficial effects of food on organ (intestine in this case) physiology.

In summary, we designed a second system that would allow continuous feeding of *C*. *elegans* with *B*. *subtilis* bacteria that have been engineered to produce dsRNA against various worm target genes. As proof of principal, we tested this RNAi system to produce animals with loss-of-function phenotypes for a small number of target genes previously shown to be involved in aging. These experiments showed first the effectiveness of this alternative method, and second, highlighted phenotypic differences that depend on which bacterial host is being used to deliver the dsRNAs molecules. Lastly, This system can be easily adapted to produce large libraries for genome wide studies, and offers the new possibility to perform RNAi manipulation in *C*. *elegans*, using an alternative food source.

In the aging research, this opens up the exiting opportunity to study this process in a less harmful and less pathogenic environmental condition; potentially allowing the discovery of new aging mechanisms went unnoticed before.

## Supporting Information

S1 Fig Analysis of *C*. *elegans* life span when grown on different bacteria as food source. Lifespan curves of animals continuously grown on one food source (A-B), or transferred from a food source to another as L4 (C-F). In all the graphs: D = Development (from L1 to L4) A = Adulthood (from L4 on). Age refers to days of adulthood. N = 70–80 worms per experiment; error bars indicate Standard Error; all experiments performed at 15°C. For further information refer also to S1 Table.
(TIF)

S2 Fig Quantification of RNAi treatment efficiency and induced phenotypes. (A) Absolute quantification of *gfp* transcript in SD1084 animals exposed to RNAi treatment vector for 48h (from L1 to L4/young adults stage at 20°C). (B) Relative quantification of *daf-2* and *glp-1* mRNA levels, in N2 animals exposed to RNAi treatment for 48h. Fold change is calculated after normalization to the expression of *tba-1* gene. Error bars indicate Standard Error of the Mean. ** = p < 0.01; * = p < 0.05 (t-test p-values). The experiment was conducted on three biological repeats. (C) Summary table of the RNAi-induced phenotypes. * = Assay performed using the NL2099 strain (rrf-3 (pk1426) II), characterized by increased sensitivity to RNAi treatment compered to wt N2 strain. (D) The quantification of DAF-16::GFP nuclear localization was performed by calculating a percentage of the worms displaying nuclear localization of DAF-16::GFP. 0—non nuclear localization in the worm; 1–5—DAF-16::GFP was observed in just a few nuclei (1–5); >10—DAF-16::GFP was observed in more then 10 nuclei per worm.
(TIF)

S3 Fig Regulation of *sod-3* expression by *unc-62*. Quantification of levels of *sod-3*::GFP expression from 20 worms in control and *unc-62* (RNAi) mutants at three time points during aging. For direct comparison in this time course experiment worms were grown and treated with RNAi expressed in (A) *E*. *coli* and (B) *B*. *subtilis*. Expression levels were determined in the head and in the intestinal area of the worm by measuring pixel intensity from GFP images. Error bars represent Standard Error of the Mean pixel intensities. *-p-value < 0.05 and ***-p-value <0.001 (t-test p-values).
(TIF)

S4 Fig *unc-62* RNAi treatment has a different effect on lipofuscin formation in worms grown on *E*. *coli* vs. *B*. *subtilis*. (A) Representative photographs of lipofuscin in wild type and *unc-62* (RNAi) animals intestines grown on *E*. *coli* or *B*. *subtilis* at day 3 of adulthood (excitation filter 360nm, emission 420nm [47]). *unc-62* (RNAi) results in decreased autofluorescence when worms are grown on *E*. *coli* as a food source, whereas *unc-62* dsRNA treatment of animals grown on *B*. *subtilis* causes induction of gut autofluorescence. (B) Quantification of levels of gut autofluorescence from 20 worms in wild type and *unc-62* (RNAi) mutants at day 3 of adulthood. Expression levels were determined in the intestinal area of the worm by measuring pixel intensity from CFP images. Error bars represent the Standard Error of the Mean pixel intensities. **-p-value < 0.01 and ***-p-value <0.001 (t-test p-values). Scale bar = 50 **μ**m.
(TIF)

S1 Table Summary of all the lifespan experiments performed in this study. Median and max survival are expressed in days. All the experiments were performed at 15°C. For further information refer also to S1 Fig. # = log-rank (Mantel-Cox) p-values; ^ = RNAi host bacterial strain; **** = p < 0.0001; *** = p < 0.001; ns = not significant.
(DOCX)
