# Supplementary figures and images for: Specific RNA Interference in Caenorhabditis elegans by Ingested dsRNA Expressed in Bacillus subtilis
Source: PLoS One. 2015 Apr 30;10(4):e0124508. doi: 10.1371/journal.pone.0124508 (PMC4416053; doi:10.1371/journal.pone.0124508)

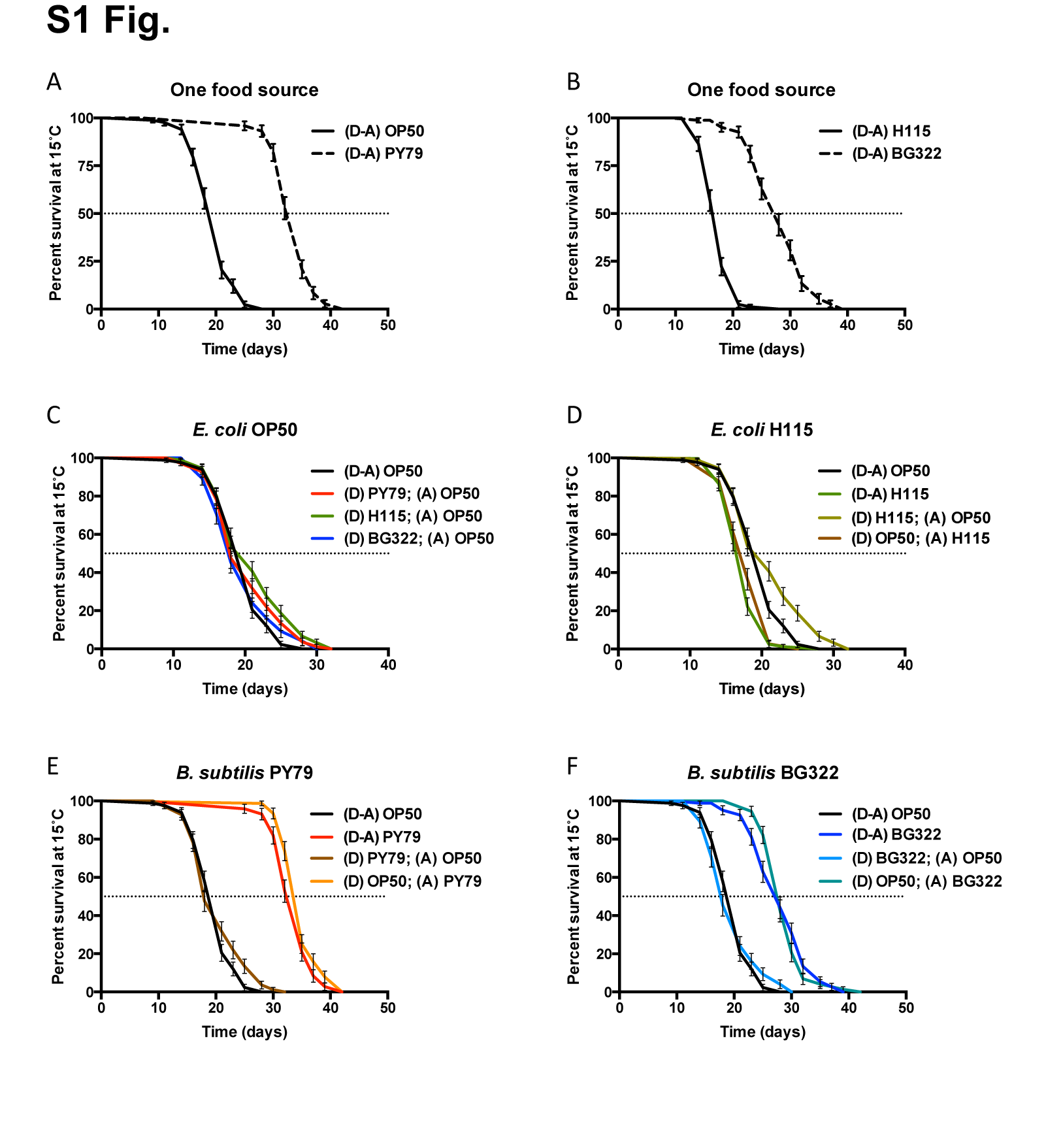

Supplement: S1 Fig — Lifespan curves of animals continuously grown on one food source (A-B), or transferred from a food source to another as L4 (C-F). In all the graphs: D = Development (from L1 to L4) A = Adulthood (from L4 on). Age refers to days of adulthood. N = 70–80 worms per experiment; error bars indicate Standard Error; all experiments performed at 15°C. For further information refer also to S1 Table. (TIF) [file pone.0124508.s001.tif]

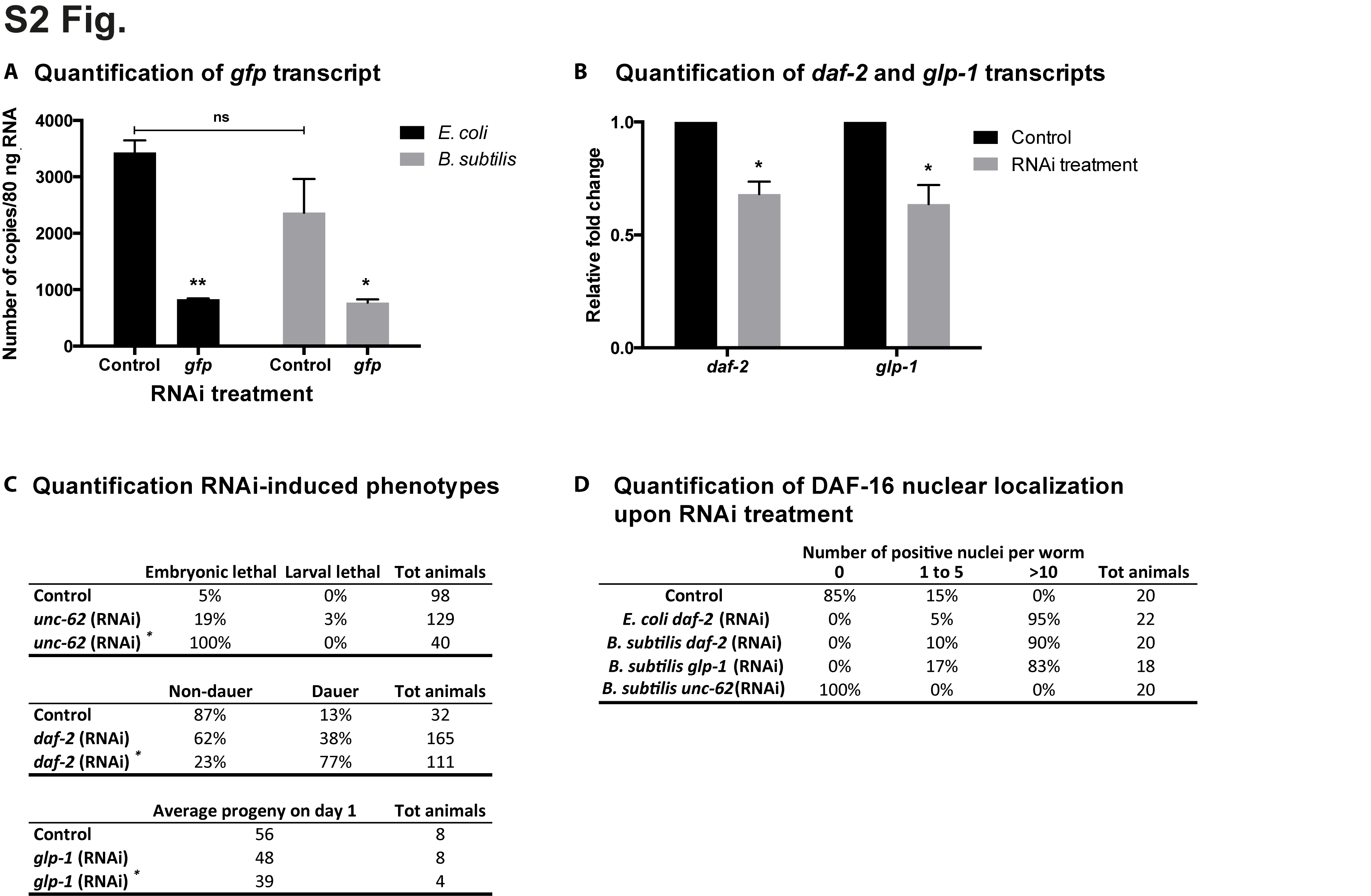

Supplement: S2 Fig — (A) Absolute quantification of gfp transcript in SD1084 animals exposed to RNAi treatment vector for 48h (from L1 to L4/young adults stage at 20°C). (B) Relative quantification of daf-2 and glp-1 mRNA levels, in N2 animals exposed to RNAi treatment for 48h. Fold change is calculated after normalization to the expression of tba-1 gene. Error bars indicate Standard Error of the Mean. ** = p < 0.01; * = p < 0.05 (t-test p-values). The experiment was conducted on three biological repeats. (C) Summary table of the RNAi-induced phenotypes. * = Assay performed using the NL2099 strain (rrf-3 (pk1426) II), characterized by increased sensitivity to RNAi treatment compered to wt N2 strain. (D) The quantification of DAF-16::GFP nuclear localization was performed by calculating a percentage of the worms displaying nuclear localization of DAF-16::GFP. 0—non nuclear localization in the worm; 1–5—DAF-16::GFP was observed in just a few nuclei (1–5); >10—DAF-16::GFP was observed in more then 10 nuclei per worm. (TIF) [file pone.0124508.s002.tif]

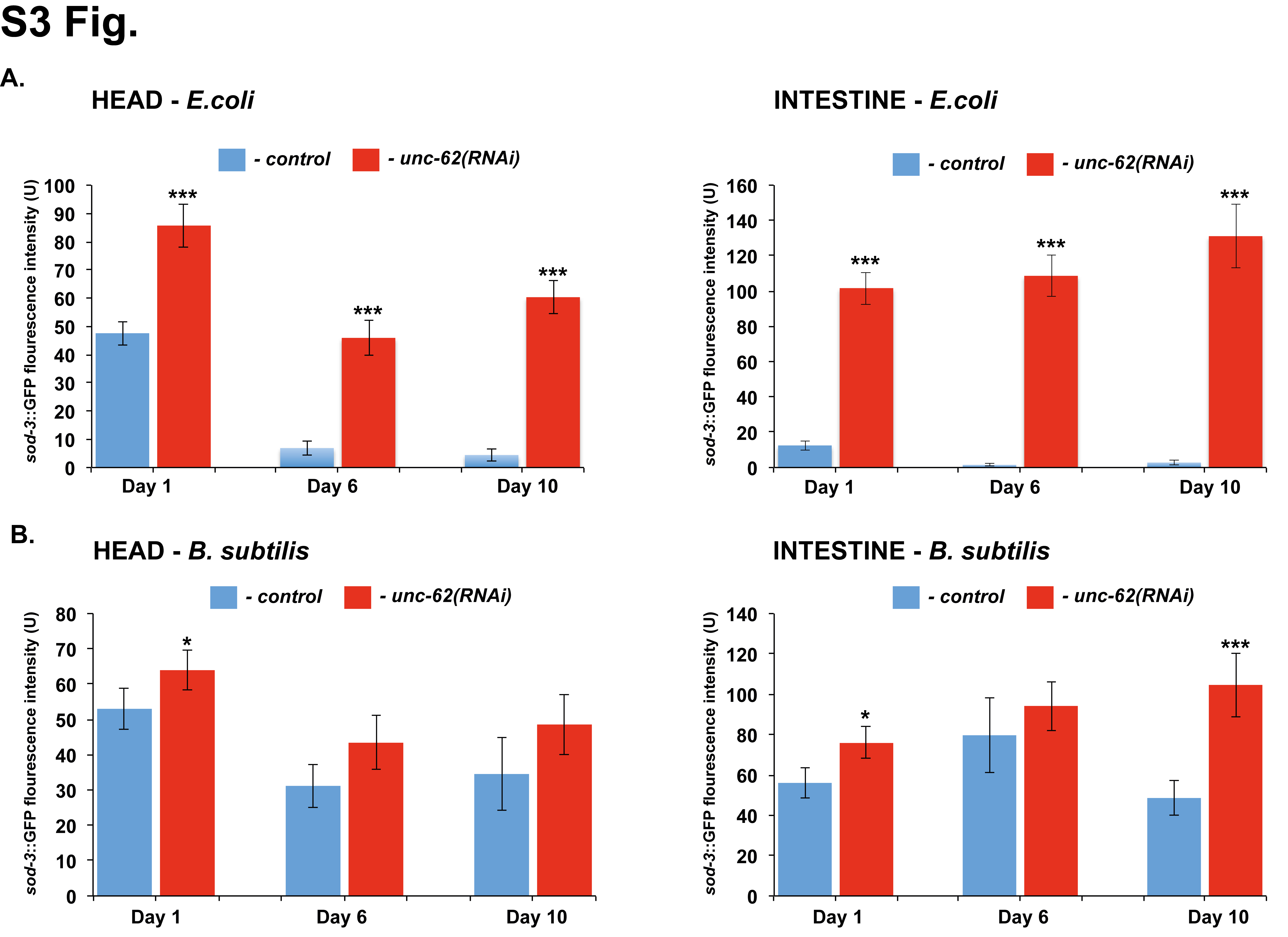

Supplement: S3 Fig — Quantification of levels of sod-3::GFP expression from 20 worms in control and unc-62 (RNAi) mutants at three time points during aging. For direct comparison in this time course experiment worms were grown and treated with RNAi expressed in (A) E. coli and (B) B. subtilis. Expression levels were determined in the head and in the intestinal area of the worm by measuring pixel intensity from GFP images. Error bars represent Standard Error of the Mean pixel intensities. *-p-value < 0.05 and ***-p-value <0.001 (t-test p-values). (TIF) [file pone.0124508.s003.tif]

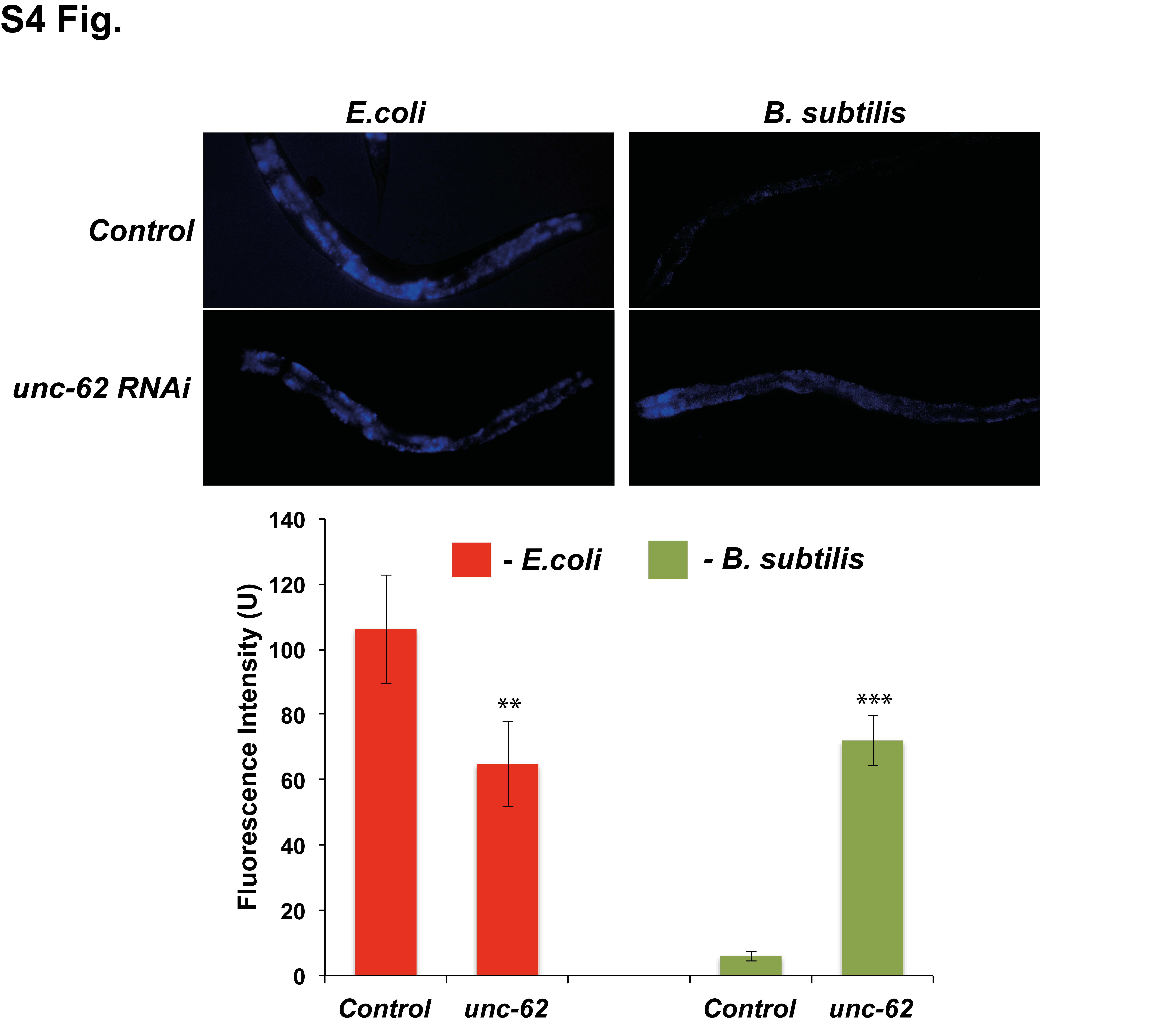

Supplement: S4 Fig — (A) Representative photographs of lipofuscin in wild type and unc-62 (RNAi) animals intestines grown on E. coli or B. subtilis at day 3 of adulthood (excitation filter 360nm, emission 420nm [47]). unc-62 (RNAi) results in decreased autofluorescence when worms are grown on E. coli as a food source, whereas unc-62 dsRNA treatment of animals grown on B. subtilis causes induction of gut autofluorescence. (B) Quantification of levels of gut autofluorescence from 20 worms in wild type and unc-62 (RNAi) mutants at day 3 of adulthood. Expression levels were determined in the intestinal area of the worm by measuring pixel intensity from CFP images. Error bars represent the Standard Error of the Mean pixel intensities. **-p-value < 0.01 and ***-p-value <0.001 (t-test p-values). Scale bar = 50 μm. (TIF) [file pone.0124508.s004.tif]
