# Supplementary material for: Specific RNA Interference in Caenorhabditis elegans by Ingested dsRNA Expressed in Bacillus subtilis
Source: PLoS One. 2015 Apr 30;10(4):e0124508. doi: 10.1371/journal.pone.0124508 (PMC4416053; doi:10.1371/journal.pone.0124508)
Supplement: S1 Table — Median and max survival are expressed in days. All the experiments were performed at 15°C. For further information refer also to S1 Fig. # = log-rank (Mantel-Cox) p-values; ^ = RNAi host bacterial strain; **** = p < 0.0001; *** = p < 0.001; ns = not significant. (DOCX) [file pone.0124508.s005.docx]

**S1 Table: Summary of all the lifespan experiments performed in this study.**

Median and max survival are expressed in days. All the experiments were performed at 15˚C. For further information refer also to S1 Fig.

# = log-rank (Mantel-Cox) p-values; ^ = RNAi host bacterial strain; **** = p < 0.0001; *** = p <0.001; ns = not significant.
